# Lateral pressure-mediated protein partitioning into liquid-ordered/liquid-disordered domains

**DOI:** 10.1039/c6sm00042h

**Published:** 2016-02-26

**Authors:** Moritz Frewein, Benjamin Kollmitzer, Peter Heftberger, Georg Pabst

**Affiliations:** a University of Graz, Institute of Molecular Biosciences, Biophysics Division, NAWI Graz, Humboldtstr. 50/III, A-8010 Graz, Austria; b BioTechMed-Graz, A-8010 Graz, Austria; c Infineon Technologies Austria AG, Development Center Graz, Babenbergerstr. 10, A-8010 Graz, Austria

## Abstract

We have studied the contributions of stored elastic energies in liquid-ordered (Lo) and liquid-disordered (Ld) domains to transmembrane proteins using the lateral pressure concept. In particular we applied previously reported experimental data for the membrane thickness, intrinsic curvature and bending elasticities of coexisting Lo/Ld domains to calculate whether proteins of simple geometric shapes would preferentially diffuse into Lo or Ld domains and form oligomers of a certain size. For the studied lipid mixture we generally found that proteins with convex shapes prefer sorting to Ld phases and the formation of large clusters. Lo domains in turn would be enriched in monomers of concave shaped proteins. We further observed that proteins which are symmetric with respect to the bilayer center prefer symmetric Lo or Ld domains, while asymmetric proteins favor a location in domains with Lo/Ld asymmetry. In the latter case we additionally retrieved a strong dependence on protein directionality, thus providing a mechanism for transmembrane protein orientation.

## 1 Introduction

For several decades lipid-only membranes have served as chemically well-defined mimics of biological membranes enabling detailed physicochemical and biophysical studies of diverse structural and dynamical membrane properties.^1^–^3^ One aspect that has ever attracted significant scientific attention is the coupling of membrane properties to protein function.

These interactions can be divided into specific lipid–protein interactions, where lipids interact with either given protein binding sites or grooves,^4^–^7^ and unspecific interactions, mediated by the membranes’ elastic and structural properties.^8^–^15^ Furthermore, peripheral membrane proteins may act as scaffolds for the global membrane curvature.^12^,^16^ For flat bilayers, hydrophobic matching is one of the most frequently discussed unspecific lipid–protein interactions, relating to the energy needed either to stretch or compress membranes near protein inclusions to alleviate thickness differences with the protein’s hydrophobic length.^11^,^17^,^18^ Alternatively, a mechanical coupling to the lateral pressure profile^10^,^19^,^20^ or stored intrinsic lipid curvatures^21^ has been considered. Importantly, any of the above discussed interactions may affect the protein function through changes in its conformational equilibrium and/or its preferred partitioning into a given lipid environment.

Here we focus on membrane-mediated protein-sorting into liquid-ordered (Lo) or liquid-disordered (Ld) domains of flat, tension-free bilayers. It is well-established that cholesterol-containing mixtures of high-melting and low-melting lipids display Lo/Ld phase coexistence over a broad range of compositions and temperatures.^22^–^24^ These systems serve commonly as models for outer plasma membranes that can be studied by an array of biophysical techniques. For example our laboratory has recently reported detailed *in situ* values for the domains’ structural and elastic properties using small-angle X-ray scattering.^25^–^27^

Differences in domain thickness have been applied to explain protein sorting based on hydrophobic matching.^28^–^30^ However, it has also been demonstrated that hydrophobic matching cannot be the unique driving force for protein partitioning into Lo or Ld domains. In particular transmembrane peptides designed to match the thickness of either Lo or Ld domains were consistently reported to be primarily localized in Ld domains.^31^–^34^ Moreover, single-membrane-spanning raft proteins were reported to partition into raft-like domains in vesicles prepared from plasma membranes, but not into Lo domains of a ternary lipid mixture.^35^

Three additional factors can be considered to resolve the disparity with protein partitioning into highly ordered phases: (i) specific interactions with lipid factors such as *e.g*. raft gangliosides,^36^ (ii) protein palmitoylation,^36^,^37^ or (iii) distinct elastic properties or lipid packing densities of a given domain.^33^,^34^

In this report we consider the latter mechanism motivated by the availability of a theoretical framework and corresponding experimental data. Specifically, the lateral pressure mechanism^19^,^20^,^38^,^39^ allowed us to calculate energetic contributions to protein partitioning as a function of overall protein size, shape, and oligimerization state by applying experimental values for Lo/Ld domain properties such as thickness, intrinsic curvature, bending rigidity, and Gaussian modulus of curvature,^25^–^27^ which are integral parameters of the lateral pressure profile.

We found that convex-shaped proteins generally prefer Ld domains, while concave-shaped proteins would sort into Lo domains. These dependencies are amplified upon increasing protein size. For proteins with cone-like shapes no significant energy gain was found to diffuse from Lo to Ld domains or *vice versa*. Furthermore, we discuss the effects of lateral pressure differences in Lo and Ld domains on protein oligomerization. Here, pressures favor the aggregation of convex-shaped proteins, while concave proteins would preferentially occur as monomers.

## 2 Methods

### 2.1 Partitioning of single proteins into Lo/Ld domains

The ratio of the occupation probabilities, or molar fractions *X*_1,2_, of two realizable states 1, 2 in a protein’s phase state, which could differ in *e.g*. protein conformation or lipid environment, is given by the partitioning coefficient *k*_P_. In thermal equilibrium, *k*_P_ solely depends on the thermal energy *k*_B_*T* and the difference Δ*W* of the states’ energy levels *W*_1,2_ and is given by^19^

(1) $$k_{\text{P}}\equiv \frac{X_{1}}{X_{2}}=\text{exp}\left(-\frac{W_{1}-W_{2}}{k_{\text{B}}T}\right)=\text{exp}\left(-\Delta W/k_{\text{B}}T\right).$$

In what follows, we consider states of different lipid environments, corresponding to Lo and Ld. For convenience we will discuss our results with respect to the natural logarithm of *k*_P_, *i.e*. −Δ*W*/*k*_B_*T*. Negative values of this term therefore reflect preferred partitioning into Ld phases and *vice versa* for positive ln *k*_P_. Furthermore, because *X*_Lo_ + *X*_Ld_ = 1 we can calculate the equilibrium concentration of proteins in the Lo phase using *X*_Lo_ = *k*_p_/(1 + *k*_p_).

**Fig. 1:**
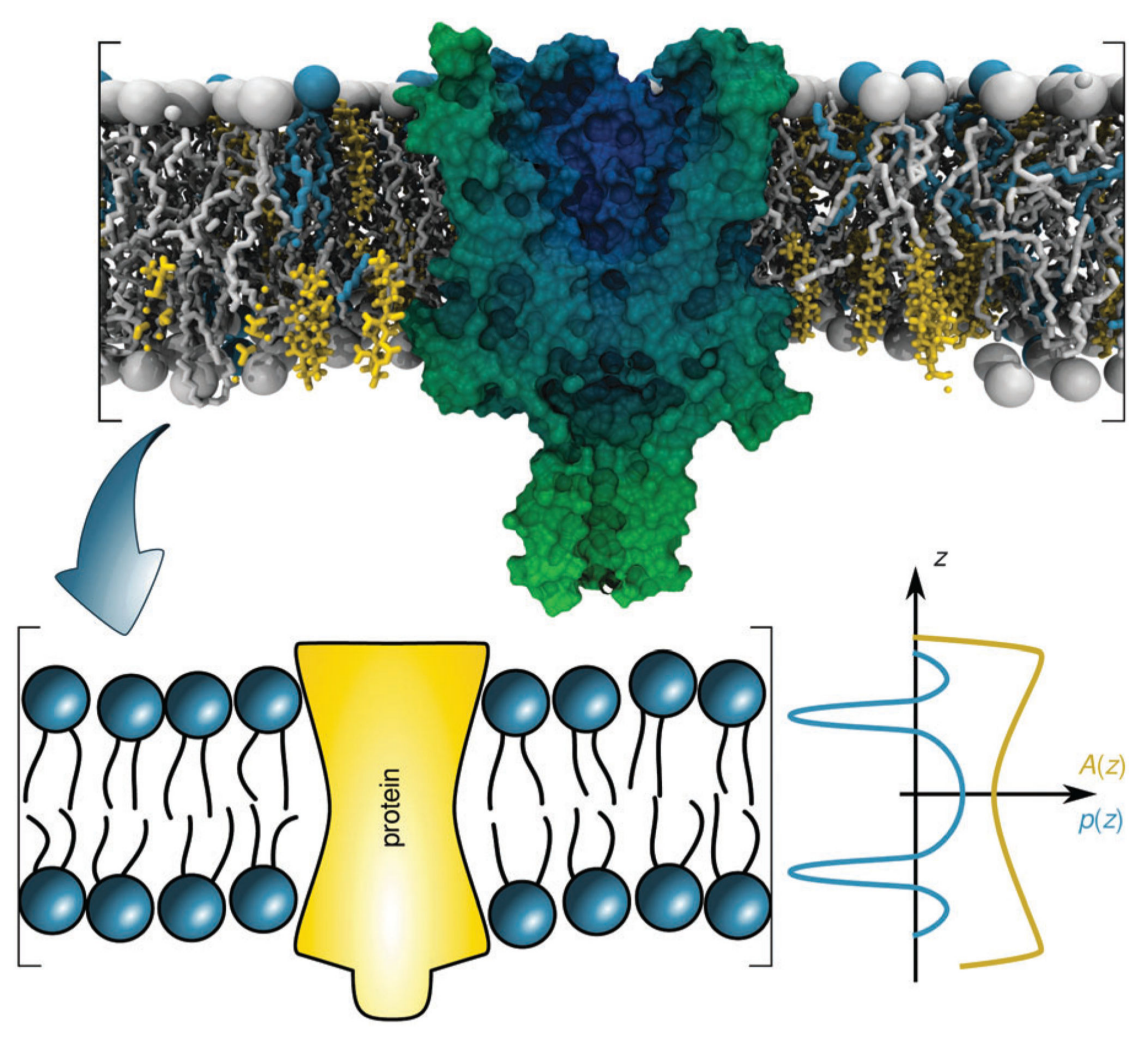
Schematic overview of the lateral pressure profile *p*(*z*) and its coupling to a membrane protein, where *z* is the coordinate normal to the bilayer surface. For calculations the complex shape of a membrane protein is transferred into a simple rotationally symmetric body with cross sectional area *A*(*z*). The molecular view on the top has been created using the CHARMM-GUI membrane builder for a mixture of distearoyl phosphatidylcholine, dioleoyl phosphatidylcholine and cholesterol in combination with a mechanosensitive channel (PDB File: 2OAR).

The transfer energy Δ*W* can depend on various contributions, *e.g*. hydrophobic matching,^28^ or lateral pressures.^10^ Here we focus on the latter mechanism. The lateral pressure profile *p*(*z*) is known to emerge from the amphiphilic properties of membrane lipids and the free energy associated with minimizing contact of the apolar regions with the aqueous phase (Fig. 1).^40^,^41^ The lateral pressure profile is difficult to determine experimentally.^42^ Thus, either mean-field theories with a lattice model for the hydrocarbon chains^19^,^20^ or molecular dynamics (MD) simulations of diverse kinds have been performed (for review see, *e.g*. ref. 43). Here we take an alternative approach that allows us to use experimental data.

The energy stored for a protein in a given lateral pressure field can be written as^19^,^20^

(2) $$W_{1,2}=\int_{-d_{\text{B}}/2}^{d_{\text{B}}/2}A(z)p(z)\text{d}z,$$

where *d*_B_ is the membrane thickness and *A*(*z*) the variation of the protein’s cross sectional area along the bilayer normal *z*. Following,^38^ we can simplify eqn (2) by expanding the protein’s cross section into a Taylor series $\left(A(z)=a_{0}+a_{1}^{\pm }\left|z\right|+a_{2}^{\pm }{\left|z\right|}^{2}+\ldots \right)$ to

(3) $$W_{1,2}=\sum_{j}a_{j}^{\pm }p_{j},$$

where ± refers to the upper or lower monolayer, respectively and $p_{j}=\int_{0}^{d_{\text{B}}/2}z^{j}p(z)\text{d}z$ is the *j*-th moment of the pressure profile. The zero’th moment gives the surface tension, which vanishes for flat, tension-free bilayers. The first and second integral moments have been shown to be^10^

(4) $$p_{1}=J_{0}\kappa_{\text{C}}$$

(5) $$p_{2}=2\kappa_{\text{C}}J_{0}h-\kappa_{\text{G}},$$

where *J*_0_ is the intrinsic lipid curvature, *κ*_C_ the monolayer bending rigidity, *h* the location of the neutral plane with respect to the center of the bilayer and *κ*_G_ the monolayer Gaussian curvature modulus. *p*_1_ is a measure for the lateral torque tension.^44^ All these parameters are experimentally accessible, see *e.g*. ref. 25–27; for *κ*_G_ we use the suggested approximation *κ*_G_ ≈ −0.8*κ*_C_.^10^

**Fig. 2:**
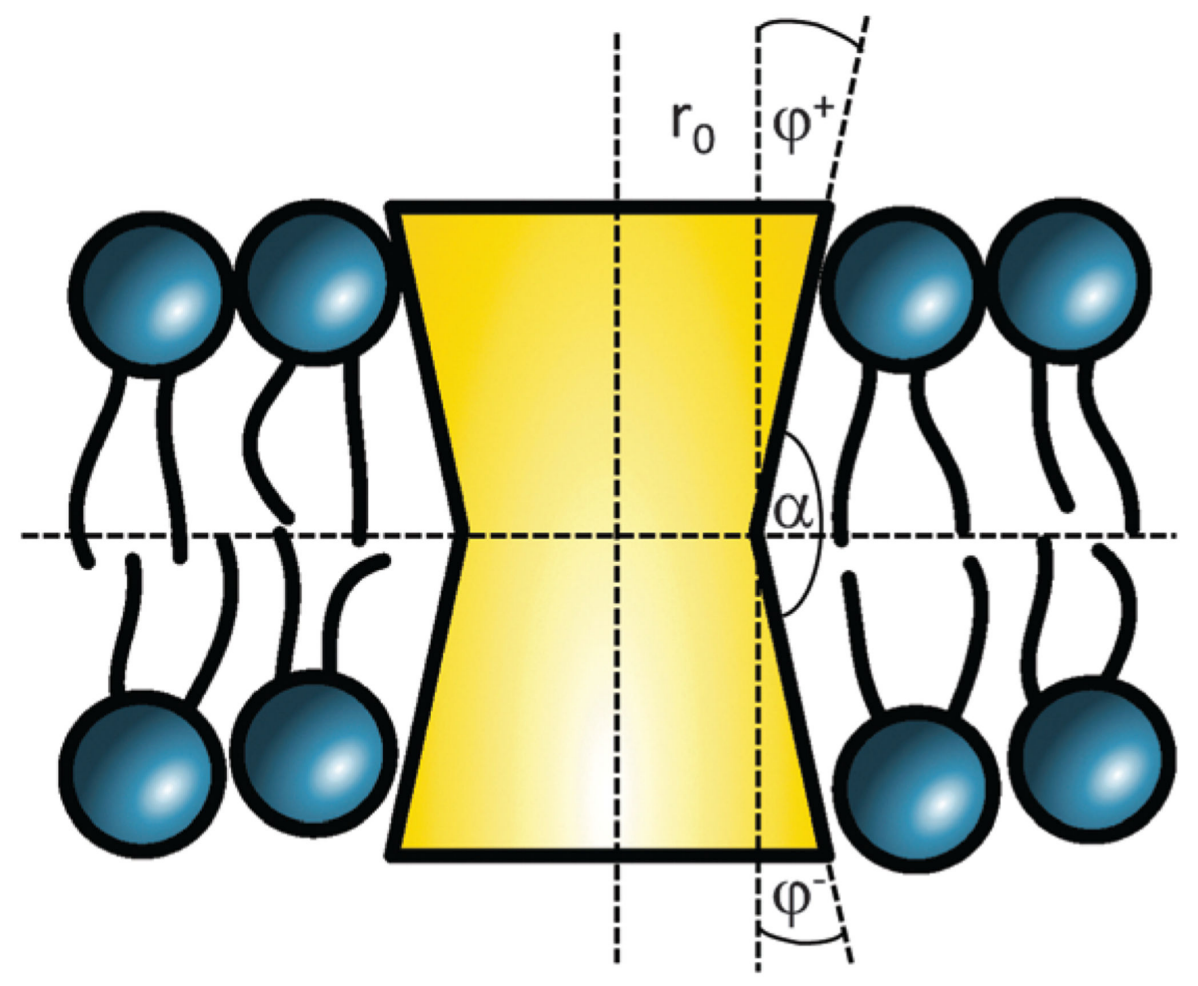
Parameterization of linear-shaped proteins. Note that cone-shaped proteins are achieved for *φ*^+^ = −*φ*^−^.

The cross sectional area of rotationally symmetric proteins *A*(*z*) = π*r*^2^(*z*) depends only on its radius *r*(*z*), and diverse shapes can be modeled using *r*(*z*) = (*r*_0_ + |*z*| tan *φ*^±^), see also Fig. 2. The protein area’s Taylor coefficients are then given by

(6) $$a_{1}^{\pm }=2\pi r_{0}\tan\varphi^{\pm },\;a_{2}^{\pm }=\pi \;\tan^{2}\varphi^{\pm }.$$

Slightly more complex shapes with smooth contour variations, see *e.g*. Fig. 1, can be achieved upon free variation of the Taylor coefficients. The energy for partitioning into a given domain is consequently calculated as $\Delta W=\sum_{j}a_{j}^{\pm }\Delta p_{j}.$

### 2.2 Formation of protein oligomers

**Fig. 3:**
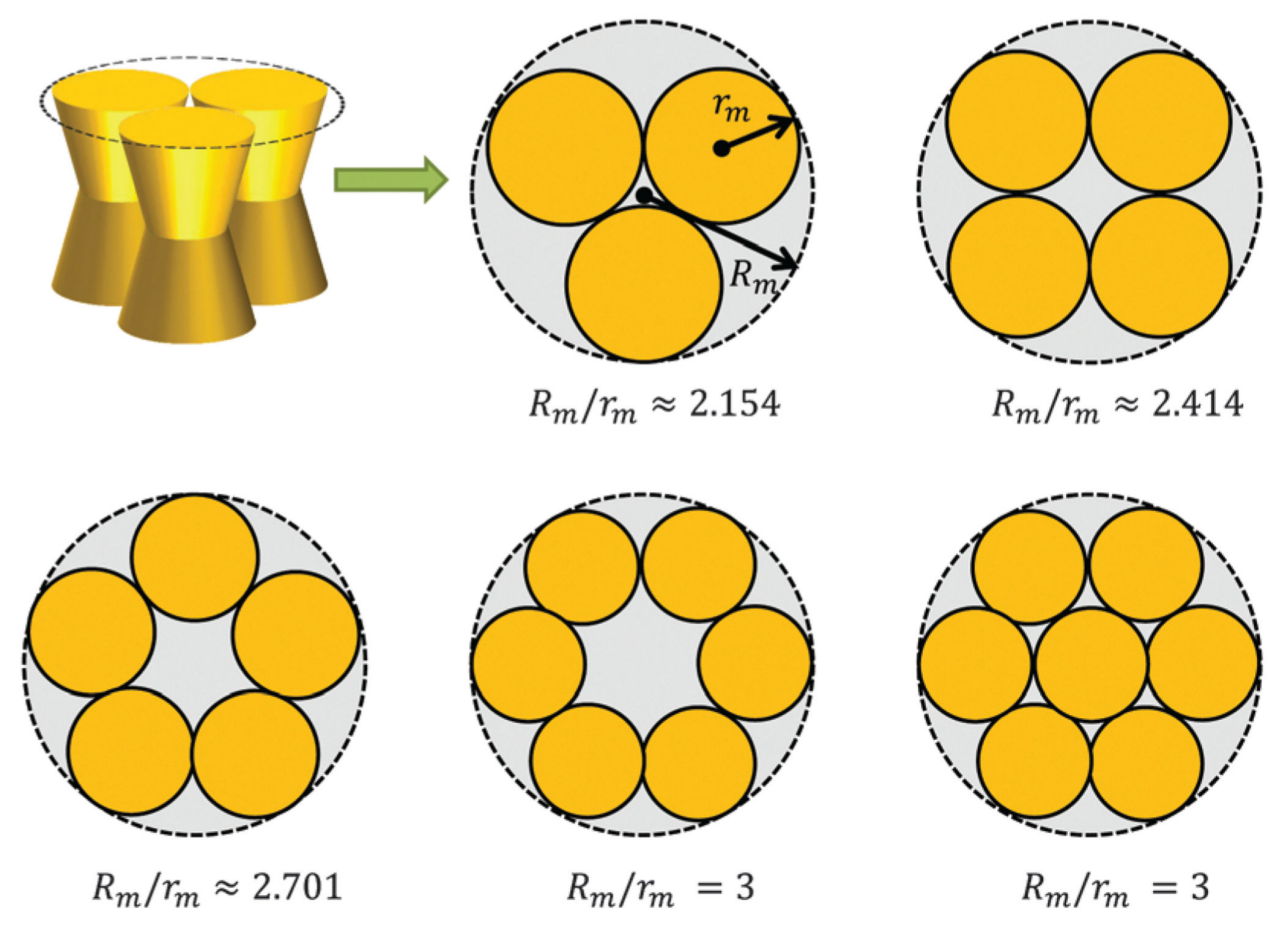
Modeling protein aggregation by dense packing of congruent circles in a circle. Here *r*_m_ is the maximum outer radius of a protein monomer of a given shape and *R*_m_ the maximum radius of a densely packed aggregate. *R*_m_/*r*_m_ ratios were taken from ref. 45.

A first-order approximation for the aggregation of *n* > 2 proteins can be achieved mathematically by considering dense packing of congruent circles in a circle.^45^ The proteins’ maximum radii *r*_m_ thus determine the maximum radius *R*_m_ of a densely packed aggregate, see Fig. 3. A cluster’s radius is then given by *R*(*z*) = *R*_m_ − *r*_m_ + *r*(*z*) and its area by *A_n_*(*z*) = π*R*^2^(*z*). Consistently, substituting *R*_0_ ≡ *R*(0) for *r*_0_ in eqn (6) gives the Taylor coefficients of *A_n_*(*z*).

To study the influence of lateral pressure on protein clustering, we are considering the changes in the protein area Δ*A*(*z*) = *A_n_*(*z*) – *nA*_1_(*z*), which equals for a given *z* the grey shaded area shown in Fig. 3, while *p*(*z*) remains constant. The difference in stored energy between an *n*-mer and *n* monomers is thus determined by $\Delta W_{n}=\sum_{j}\Delta a_{j}^{\pm }p_{j},$ where $\Delta a_{j}^{\pm }$ denotes the Taylor coefficients of Δ*A*(*z*). In equilibrium the partitioning coefficient is then defined as^39^

(7) $$k_{\text{P},n}=\frac{X_{n}}{X_{1}^{n}}=\text{exp}\left(-\frac{\Delta W_{n}}{k_{\text{B}}T}\right).$$

Calculation of the protein concentration in an *n*-mer aggregate leads to

(8) $$X_{n}-k_{\text{P},n}{\left(1-X_{n}\right)}^{n}=0,$$

which can be solved numerically.

## 3 Results

For the present calculations we applied structural data for coexisting Lo/Ld domains in a ternary mixture of dioleoyl phosphatidylcholine (DOPC) distearoyl phosphatidylcholine (DSPC) cholesterol (Chol) reported from X-ray scattering experiments.^25^–^27^ For completeness data are summarized in Table 1. The differences between Lo and Ld structural and elastic properties, discussed in detail in our previous reports, lead to distinct values for the first and second lateral pressure moments. Most significantly, *p*_2_ changes its sign from Ld to Lo, which is mainly due to the more negative intrinsic curvature of the Lo phase and its increased thickness (*h*_Lo_ > *h*_Ld_).

In the following we will first present the effects of these differences on the partitioning of protein monomers of different shapes and then discuss contributions of lateral pressures in Lo and Ld to protein aggregation.

### 3.1 Shape-dependence of protein partitioning

**Fig. 4:**
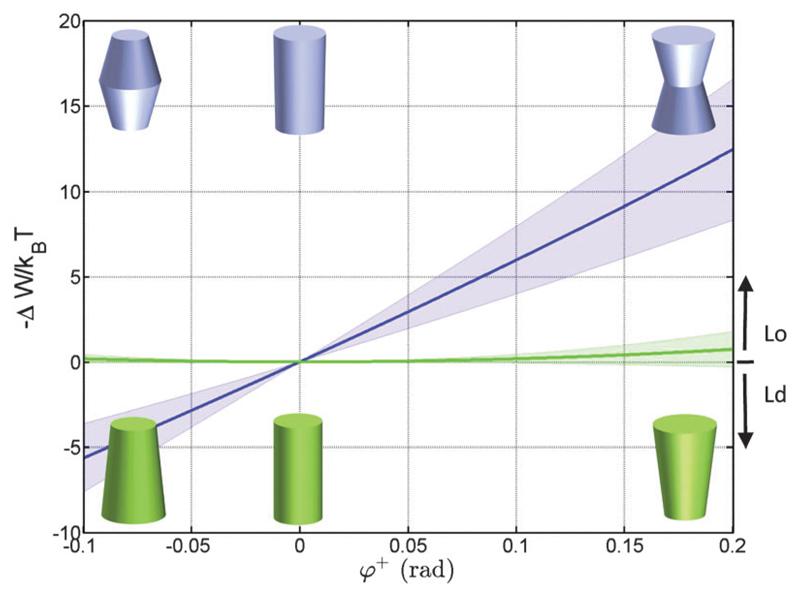
Effect of the opening angle *φ*^+^ on the partitioning of proteins into Lo (−Δ*W/k_B_T* > 0) and Ld (−Δ*W/k_B_T* < 0) domains displayed by a DOPC/DSPC/Chol mixture (*r*_0_ = 2 nm). The blue line describes the results for proteins changing their shape from convex to concave forms; the green line for cone-shaped proteins. Shaded areas indicate uncertainties due to experimental errors (Table 1).

Using the parameterization described in Fig. 2, we first calculated the partitioning coefficients for inward and outward bent proteins, varying the opening angle *φ*^+^ = *φ*^−^ (Fig. 4). Results are either symmetric or anti-symmetric with respect to cylindrically-shaped proteins, which do not exhibit preferred partitioning in Lo or Ld phases, because their shape does not act against the lateral strains stored in the bilayers. In turn, concave-shaped proteins prefer partitioning into Lo domains and convex-shaped proteins into Ld domains, respectively. This can be understood qualitatively in view of the change of the first moment in going from Ld to Lo domains Δ*p*_1_ < 0, which signifies that lateral pressures are redistributed from the lipid/water interface to the bilayer interior, thus favoring a location of inward-bent proteins in the Lo phase.

Cone-shaped proteins (*φ*^+^ = −*φ*^−^) exhibit symmetric partitioning preferences with respect to *φ*^+^ = 0 (Fig. 4) because terms linear in tan *φ*^±^ compensate due to the bilayer symmetry. Quadratic terms affect a preference of these proteins for partitioning into Lo domains. However, the involved energies are only slightly above thermal energies and consequently rather insignificant.

**Fig. 5:**
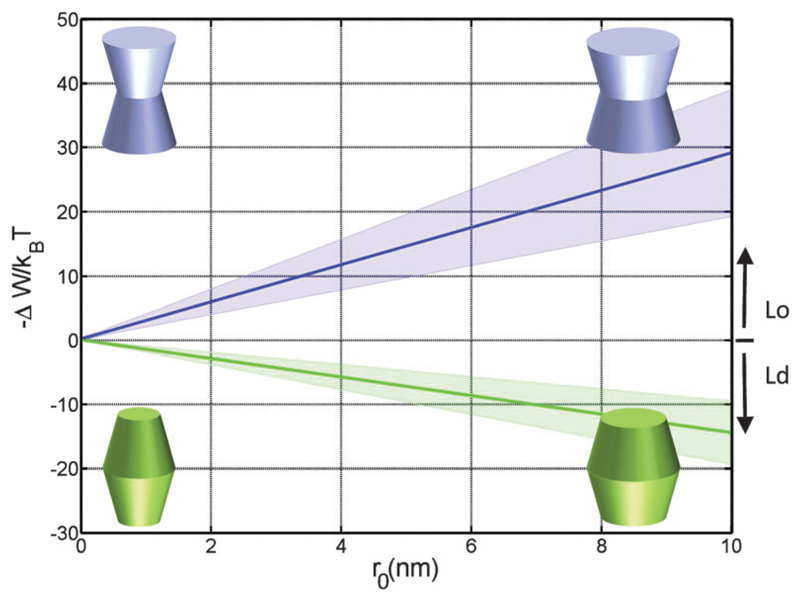
Effect of the protein size (*r*_0_) on partitioning of proteins into Lo and Ld domains of a DOPC/DSPC/Chol mixture for proteins with concave (*φ*^−^ = *φ*^+^ = 0.1 rad; blue) and convex (*φ*^−^ = *φ*^+^ = −0.05 rad; green) shapes.

Concerning size, only convex/concave proteins exhibit a distinct dependence. In particular we found a linear increase for the preference of sorting into either Lo or Ld domains (Fig. 5). Cone-shaped proteins in turn do not change their preferred sorting to Lo domains with size. This is again due to the neutralization of linear terms.

So far we have described proteins with a plane of symmetry in center of the bilayer. For |*φ*^+^| ≠ |*φ*^−^| we found similar tendencies for Lo/Ld partitioning as for symmetric proteins. This can be generalized in terms of the angle *α* describing the protein’s bending direction (Fig. 2). Proteins with *α* < π prefer sorting to Lo phases, while shapes with *α* > π would diffuse into Ld domains.

### 3.2 Influence of curvature

Using $r(z)=\sqrt{\left(a_{0}+a_{1}^{\pm }\left|z\right|+a_{2}^{\pm }{\left|z\right|}^{2}\right)/\pi }$ allows the generation of smooth protein contours. In the following we will restrict – due to symmetry – the presentation of our results to the upper half of the protein’s shape. Interestingly, proteins with significantly different shapes have equal partitioning energies for constant $r\prime (0)\left({=\frac{\text{d}r(z)}{\text{d}z}|}_{z=0}\right)$ This can be quantified upon comparison to eqn (6) through the angle

(9) $$\theta =\text{arctan}\left(\frac{a_{1}}{2\pi r\left(0\right)}\right)=\text{arctan}\left(\frac{a_{1}}{2\sqrt{a_{0}}}\right).$$

**Fig. 6:**
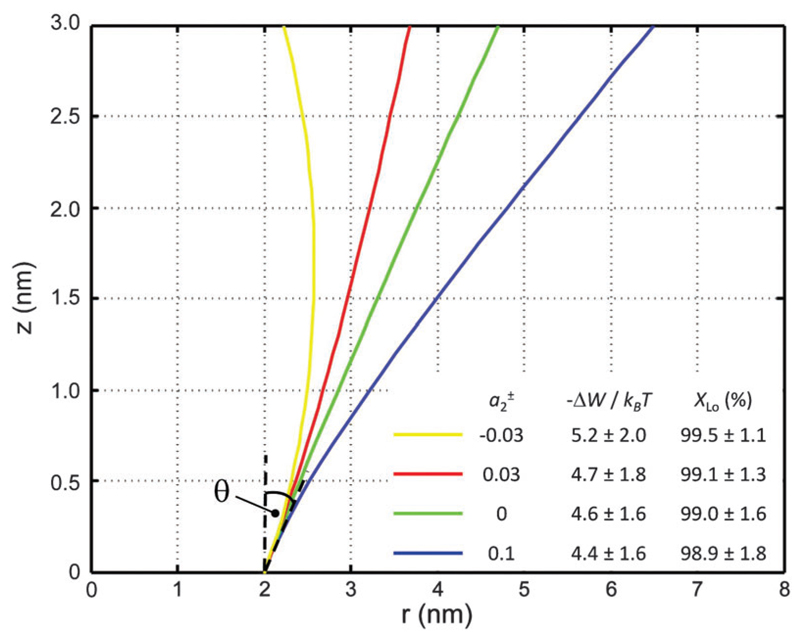
Inward-bent proteins with different contours, but similar partitioning probabilities into Lo domains (*a*_0_ = 4π nm^2^, $a_{1}^{\pm }$ = 1 nm). The inset shows the results for different $a_{2}^{\pm }$ values.

Since *a*_0_ does not contribute to *W* in tension free bilayers, varying *a*_0_, while keeping *a*_1_ and *a*_2_ constant, yields equal *k*_P_ values. Furthermore, even minor variations of *θ* induced by small changes of *a*_2_, result in comparable partitioning probabilities for Lo domains at significant different protein shapes (Fig. 6).

**Fig. 7:**
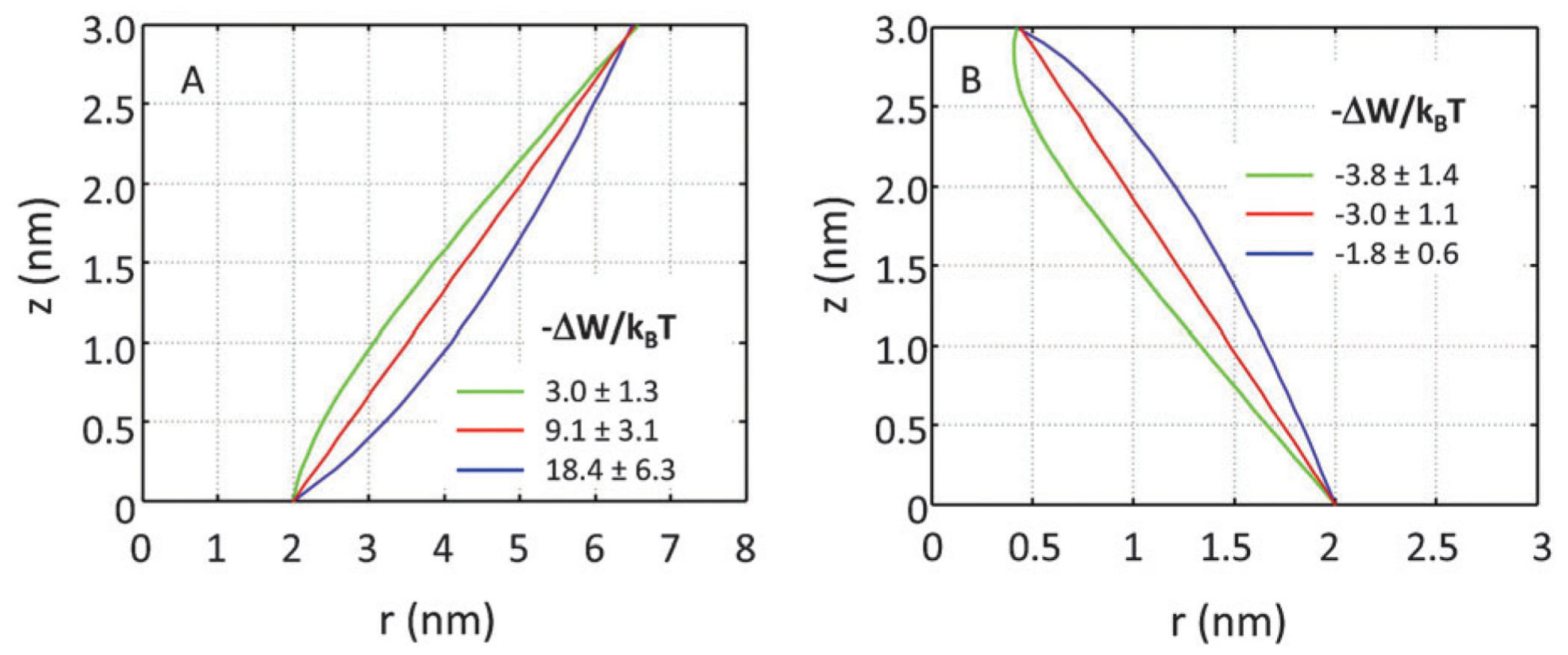
Effect of inward or outward bending of the protein’s contour for concave (panel A) and convex (panel B) proteins (*r*_0_ = 2 nm).

If *r*′(0) is allowed to vary significantly, we find strikingly different partitioning coefficients for proteins of similar dimensions. To this end, let us consider proteins under the constraint *a*_1_ + *a*_2_·*h* = const. That is, the proteins are tied to the same cross-sections at *z* = 0 and at *z* = *h*. We observed that concave-shaped proteins, see Fig. 7A, have a decreased (increased) propensity to partition into Lo domains if their contour is inward (outward) bent. Likewise, convex-shaped proteins increase (decrease) their preference for Ld phases for inward (outward) bent contours, see Fig. 7B.

### 3.3 Asymmetric domains

So far we have restricted our analysis to the simple picture of phase-separated, but symmetric membranes. Natural plasma membranes exhibit, however, a considerable degree of lipid asymmetry.^46^ Most recently protocols have become available, which enable a characterization of asymmetric model membranes with a number of biophysical techniques.^47^–^50^ It can be anticipated therefore that experimental data such as those reported in Table 1 will become available for phase separated asymmetric bilayers in due time. In the meantime it is instructive to estimate lateral pressure effects on protein partitioning assuming that Lo and Ld monolayer domains have the same properties as in symmetric bilayers.

**Fig. 8:**
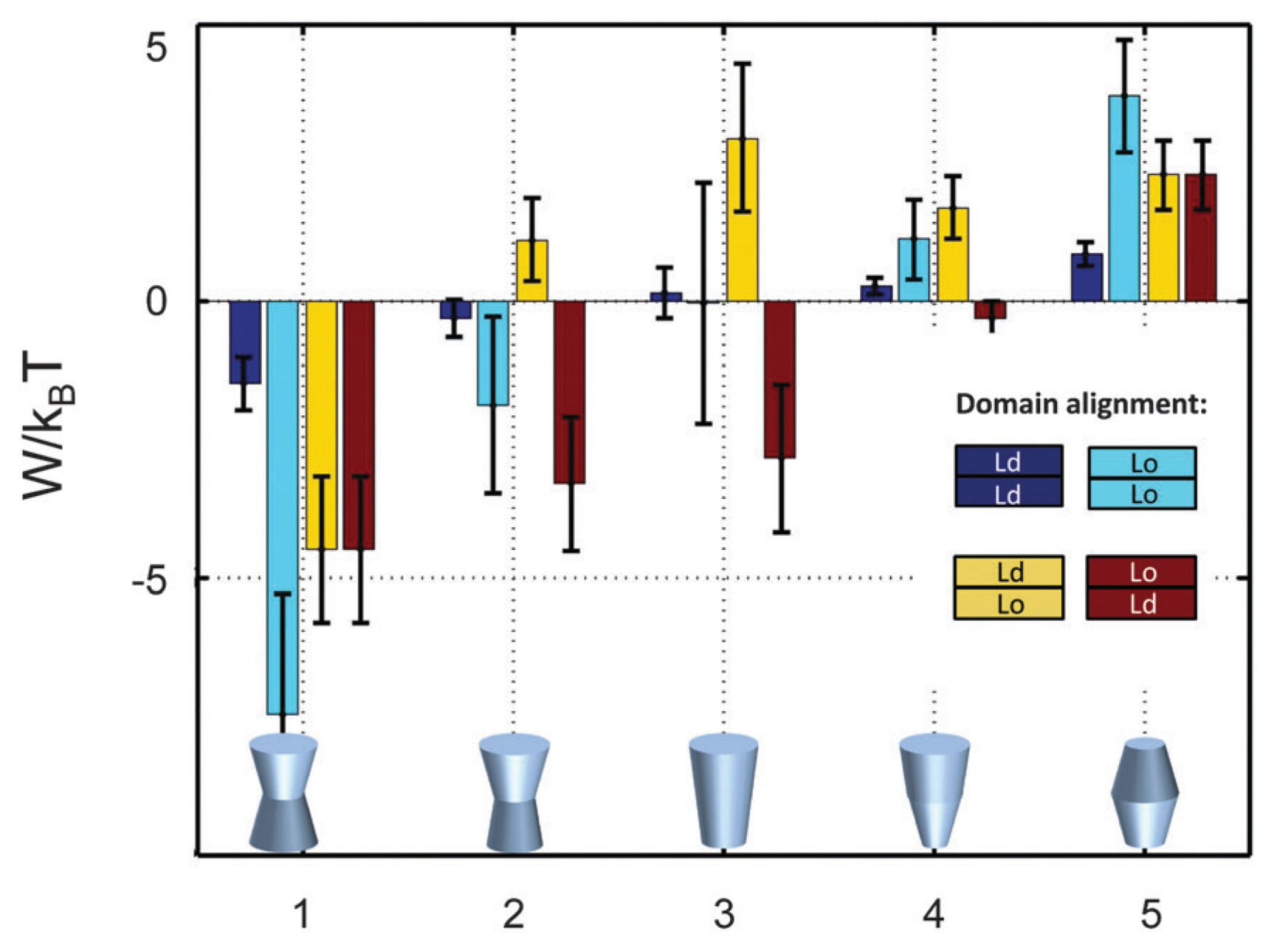
Stored lateral strain energy for proteins of different shapes in symmetric or asymmetric Lo and Ld domains. Data have been calculated using *r*_0_ =2 nm and (*φ*^+^; *φ*^−^) values of shape 1: (0.1; 0.1), shape 2: (0.1; 0.05), shape 3: (0.1; −0.1), shape 4: (0.02; −0.05) and shape 5: (−0.05; −0.05). The partitioning of a protein of given shape in a specific lipid environment is calculated through −Δ*W*/*k*_B_*T* (see (eqn (1))). The overall preferred lipid environment for a given protein shape is given by the lowest *W*/*k*_B_*T*-value.

Besides considering transbilayer correlation or anti-correlation of Lo and Ld phases, our calculations also included a variation of protein symmetry with respect to *z* = 0. Fig. 8 shows the stored elastic energies W in the different lipid environments for selected protein shapes. The preferred lipid environment exhibits the lowest stored elastic energy value for a given protein.

Intriguingly, we found that symmetric proteins (shapes 1 and 5 of Fig. 8) would sort either to symmetric Lo (concave-shaped) or Ld (convex-shaped) domains, while asymmetric proteins (shapes 2–4) favor anti-correlated domains. The sorting to anti-correlated domains depends strongly on protein orientation, however. Asymmetric proteins with larger diameters on upper membrane boundary (*r*(*h*) > *r*(−*h*)) prefer sorting to Ld^inner^/Lo^outer^ domains, while Lo^inner^/Ld^outer^ is even less energetically favorable than symmetric Lo or Ld domains. Hence, transmembrane proteins may flip horizontally within asymmetric membranes due to the lateral pressure field in order to lower their free energy. Thus, besides commonly considered contributions, such as the overall charge distribution of polar amino acid residues and ionic membrane lipids,^51^ also lateral membrane pressures provide a means to orient membrane proteins.

### 3.4 Protein oligomerization

Already early concepts for complex membrane organization considered membrane rafts as platforms for protein assembly.^52^ Recent super-resolution microscopy experiments further indicated the formation of large membrane protein clusters.^53^–^55^ Here, we consider in a highly simplified way the contributions of membrane lateral pressure to this effect using the methodology described in Section 2.2.

Our previous calculations showed the preference of concave-shaped proteins for Lo and convex-shaped proteins for Ld domains. Hence, we focus on the question whether the proteins would tend to form clusters in their preferred lipid environment or not. In particular we considered the formation of trimers to heptamers (Fig. 3).

**Fig. 9:**
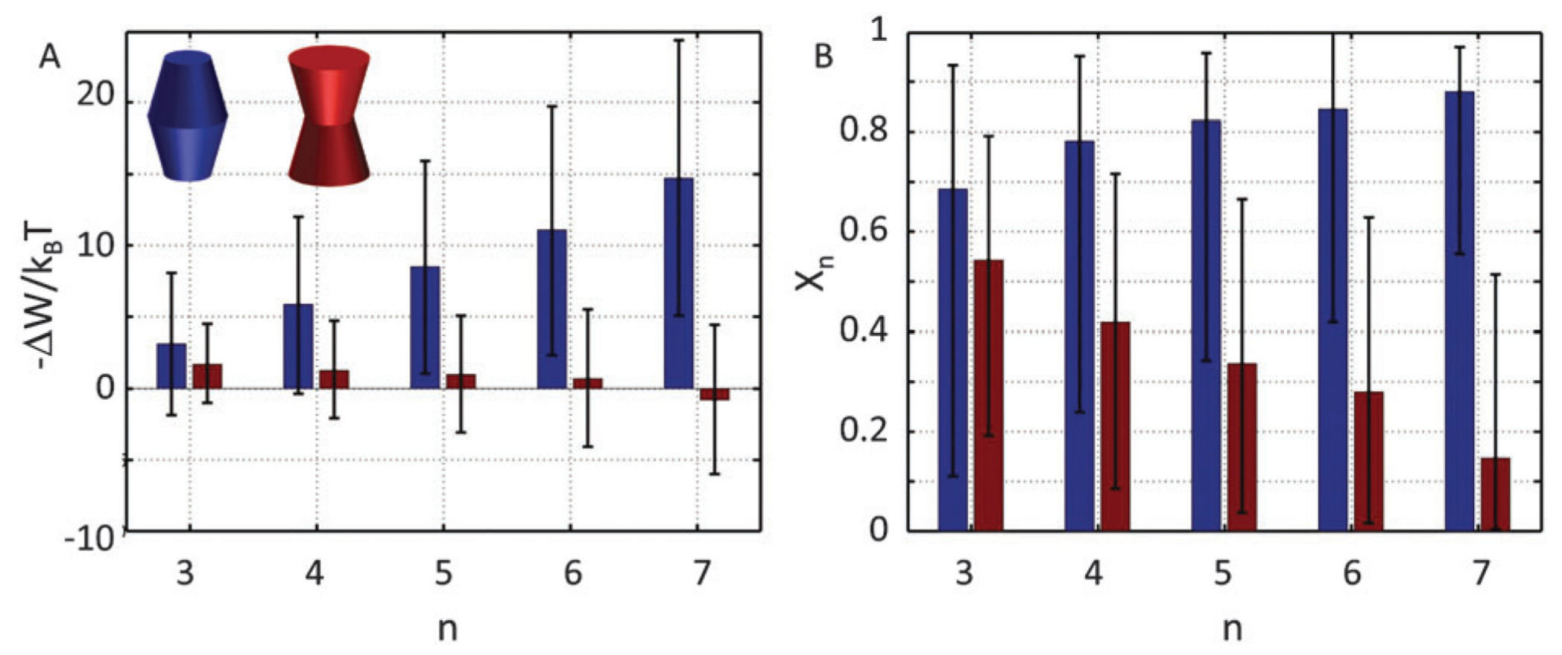
Cluster formation of convex-shaped proteins (*φ*^±^ = −0.05) in Ld domains (blue) and concave-shaped proteins (*φ*^±^ = 0.1) in Lo domains (red), using *r*_0_ = 2 nm. Panel A shows the energetic gain for cluster formation and panel B the relative concentration of aggregates of a given size *n*.

Our results demonstrate that the lateral pressure distribution in Lo domains would drive concave proteins towards monomeric forms, while convex proteins in Ld domains would tend to aggregate into large clusters (Fig. 9A). Furthermore, the fraction of clustered proteins is significantly higher for convex proteins at all aggregate sizes (Fig. 9B).

This behavior can be understood in terms of the packing differences between concave and convex proteins. Because the opening angle *φ*(=*φ*^+^ = *φ*^−^) is assumed to be the same for the monomer and aggregate, Δ*A*(*z*) = Δ*a*_1_·|*z*| = 2π tan *φ*(*R*_0_ – *nr*_0_)·|*z*| (eqn (6)). Aggregate stability requires Δ*W_n_* > 0. Since *p*_1_ < 0 for both considered lipid phases (Table 1), aggregates are stable if Δ*a*_1_ is negative as well, which is equivalent to *R*_0_/*r*_0_ < *n*. Clusters composed of convex protein monomers have the highest packing density at the center of the bilayer, i.e. they are stable if *R*_m_/*r*_m_ < *n*. This is always achieved for *n* > 2 (see ratios in Fig. 3). Oligomers of concave proteins in turn have the highest packing density at the lipid/water interface (±*d*_B_/2) and are consequently more loosely packed at the bilayer midplane. This increases their *R*_0_/*r*_0_ ratio, with respect to convex aggregates making the clusters formed of concave proteins less stable. Specifically for the *r*_0_ and *φ* values used in Fig. 9 these oligomers are unstable.

## 4 Discussion

We have studied, based on the availability of experimental data, the influence of lateral pressures on the sorting and cluster formation of transmembrane proteins. Several assumptions were made to perform these calculations.

Firstly, all considered proteins were of simple geometric shape with smooth surfaces. For more complex shapes higher lateral pressure moments would need to be defined and measured. Alternatively, MD simulations could be applied in combination with crystallographic data for membrane proteins, *e.g.* ref. 43. However, uncertainties due to limitations of simulation box size and inaccuracies in MD force fields^56^ as well as unknowns of exact protein conformation in a given lipid environment^57^ would be still rather significant. Yet, our calculations using somewhat more complex protein shapes with smoothly curved contours show that trends of preferential partitioning are conserved (Fig. 6 and 7), *i.e*. outward-bent proteins prefer Ld and inward-bent proteins Lo phases. Thus, the overall tendencies for protein sorting due to lateral pressures are captured by our simplifications. The strength of these tendencies of course depends on the exact shape and would require exact knowledge of protein conformation and *p*(*z*).

Secondly, our calculations are based on a single lipid mixture of DOPC/DSPC/Chol. It is highly conceivable that changing lipid composition may influence the here observed tendencies considerably due to changes in *h*, *J*_0_, *κ*_C_ and *κ*_G_. For example, we found previously that increasing temperature leads to a redistribution of cholesterol from Lo to Ld domains,^26^ which due to its large negative intrinsic curvature^25^ will significantly affect the domain’s elastic properties. Importantly, present calculations do not consider protein-induced modifications of structural and elastic properties of Lo and Ld phases. Previous studies on single lipid membranes demonstrated that proteins may shift these properties significantly and correlate strongly with protein concentration (see *e.g*. ref. 13). Distinct effects on Lo and Ld domains are presently unknown, but would warrant further research.

Thirdly, we neglected contributions from specific lipid/protein correlations^4^–^7^,^36^ as well as unspecific interactions such as hydrophobic matching^28^–^30^ or protein diffusion barriers at the domain boundaries which may act as local sinks for the proteins. The energies involved in these interactions are not trivial to determine. However, for single membrane-spanning peptides hydrophobic matching was not found to contribute to protein partitioning.^34^ The same authors report, however, that hydrophobic matching affects protein aggregation.

Despite all approximations and limitations discussed in the above paragraphs, the lateral pressure fields stored in Lo and Ld domains provide a fundamental contribution to protein sorting into a given lipid environment, which needs to be considered in a comprehensive picture of lipid/protein interactions in complex membranes. Our results demonstrate the preference of outward-bent proteins to the more loosely packed Ld domains, where they would tend to form clusters of large size. Our calculations did not result in an optimal aggregation size, however (Fig. 9). We speculate that this will be strongly determined by specific protein/protein or lipid/protein interactions. Inward-bent proteins, in turn preferentially locate in the more dense Lo domains in monomeric form if only contributions from lateral pressures are considered.

Qualitatively, a complementary view can be taken by considering the proteins to be rigid bodies, whose shapes affect lipid packing in their vicinity. Dan and Safran^21^ considered the free energy contributions of interfacial packing mismatches and found them to be dominated by differences in intrinsic lipid curvatures. Assuming that lipids do not demix in the vicinity of the protein inclusion this would mean that Lo phases, having a more negative *J*_0_ than Ld phases (Table 1), favor concave proteins and *vice versa* for convex proteins, which agrees with our findings.

It is interesting to compare our results to experimental findings. For example, the multitransmembrane strand protein perfringolysin O (PFO) was found to prefer sorting to Lo domains.^30^ Although authors have attributed this to hydrophobic matching, we note that PFO forms a multimeric barrel with an overall concave shape, which according to our results favors lateral pressures in Lo domains. The pentameric nicotinic acetylcholine receptor (nAChR) in turn was found to lack preference for Lo domains.^58^ Structural studies suggest a cone-like structure of nAChR’s transmembrane domain,^59^ for which shapes our calculations do not yield significant contributions for Lo partitioning. However, lateral pressures in asymmetric Ld^inner^/Lo^outer^ domains strongly favor the partitioning of such proteins (Fig. 8). This observation matches with the recent findings by Perillo *et al*.^60^ who suggested the specific interactions of nAChR with outer leaflet sphingomyelin. Thus, partitioning of both proteins could also be rationalized in terms of lateral pressures, although – in view of the many assumptions involved in our calculations – we explicitly refrain from stating that this is the only contribution driving this behavior. Lateral pressures, however, do not contribute to the sorting of single-membrane-spanning proteins due to their nearly cylindrical shape (Fig. 4) and small size (Fig. 5) within the membrane’s interior.

For asymmetric proteins in correlated or anti-correlated Lo/Ld domains we found an additional strong coupling to the protein’s preferred orientation. Thus, besides influencing protein sorting, lateral pressures also contribute to the direction of transmembrane proteins. Our results consequently allude to the importance of directional membrane-mediated protein sorting and encourage further research along these directions.

## Figures and Tables

**Table 1:** Structural and elastic data of DOPC/DSPC/Chol (0.42 : 0.37 : 0.21) at 20 °C and the corresponding lateral pressure profile moments

	Ld	Lo
*J*_0_^a^ (nm^−1^)	−0.12 ± 0.01	−0.20 ± 0.04
*h*^b^ (nm)	1.68 ± 0.03	2.12 ± 0.04
*κ*_C_^c^ (*k*_B_*T*)	5.4 ± 1.2	14.8 ± 2.5
*κ*_G_^d^ (*k*_B_*T*)	−4.4 ± 1.2	−11.9 ± 2.7
*p*_1_ (pN)	−2.6 ± 0.7	−12 ± 33
*p*_2_ (*k*_B_*T*)	2.2 ± 1.2	−0.7 ± 3.5

a Calculated from ref. 25 using (DOPC/DSPC/Chol)_Ld_ = 0.79 : 0.09 : 0.12 and (DOPC/DSPC/Chol)_Lo_ = 0.05 : 0.65 : 0.30.

b Derived from the position of the carbon glycerol groups reported in ref. 26.

c Taken from ref. 27.

d Calculated using *κ*_G_/*κ*_C_ = −0.80 ± 0.05.^10^
